# Individual Diet Modification Reduces the Metabolic Syndrome in Patients Before Pharmacological Treatment

**DOI:** 10.3390/nu13062102

**Published:** 2021-06-19

**Authors:** Małgorzata Elżbieta Zujko, Marta Rożniata, Kinga Zujko

**Affiliations:** 1 Department of Food Biotechnology, Faculty of Health Sciences, Medical University of Bialystok, Szpitalna 37, 15-295 Bialystok, Poland; 2 Department of Dietetics, Faculty of Health Sciences, Lomza State University of Applied Sciences, Akademicka 14, 18-400 Lomza, Poland; martaa.kijek@wp.pl; 3 Students’ Scientific Society, Department of Cardiology, Medical University of Bialystok, M. Sklodowskiej-Curie 24A, 15-276 Bialystok, Poland; z_kinga@wp.pl

**Keywords:** dietary habits, waist circumference, fasting glucose, high-density lipoprotein, triglycerides, blood pressure, polyphenols

## Abstract

Modification of lifestyle, including healthy nutrition, is the primary approach for metabolic syndrome (MetS) therapy. The aim of this study was to estimate how individual nutrition intervention affects the reduction of MetS components. Subjects diagnosed with MetS were recruited in the Lomza Medical Centre. The study group consisted of 90 participants and was divided into one intervention group (individual nutrition education group (INEG)) and one control group (CG). The research was conducted over 3 months. The following measurements were obtained during the first visit and after completion of the 3 months intervention: body mass, waist circumference, body composition, blood pressure, fasting glucose, and blood lipids. Dietary assessments were performed before and post-intervention using 3-day 24-h dietary recalls. Dietary knowledge was evaluated with the KomPAN questionnaire. The total polyphenol content of the diet was calculated. Sociodemographic and lifestyle characteristics were collected from a self-reported questionnaire. The physical activity was assessed by the short version of the International Physical Activity Questionnaire (IPAQ). It was found that the individual nutrition education was an effective method to improve the knowledge, dietary habits, and physical activity of the study participants. The modification of the diet in terms of higher intake of polyphenols (flavonoids and anthocyanins), fiber, polyunsaturated fatty acids (PUFA), PUFA n-3, and lower intake of saturated fatty acids (SFA) had a significant impact on the improvement of some MetS risk factors (waist circumference, fasting glucose, and HDL-cholesterol).

## 1. Introduction

The metabolic syndrome (MetS) is a complex of interrelated risk factors for the development of type 2 diabetes mellitus (T2DM) and cardiovascular disease (CVD), which are the two leading causes of death worldwide. The criteria for the diagnosis of MetS have been continuously modified over the years. In 2009, the International Diabetes Federation (IDF) and the American Heart Association/National Heart, Lung, and Blood Institute (AHA/NHLBI) adopted common criteria for MetS diagnosis, which are still relevant [1].

It is estimated that MetS is diagnosed in 20–25% of the global adult population, in 1/3 of the US population, and 1/4 of the European population [2,3,4]. Our previous study, performed in a representative sample of the Polish population in the Multicenter National Population Health Examination Survey (WOBASZ II study), showed that MetS criteria were met by 36% of participants [5].

Modification of lifestyle, including healthy nutrition and physical activity, is the primary approach for MetS therapy. Several previous studies have reported that high-quality dietary patterns may reduce the risk of MetS in different populations [6,7,8]. Other studies have underlined the role of bioactive components (such as polyphenols) and bioactive-enriched food in MetS reduction [9,10].

Dietary polyphenols are naturally occurring compounds found in plants (mainly fruits, vegetables, tea, coffee, herbs, and nuts). Polyphenols are a large and diverse group that can be divided into four major classes including: flavonoids, phenolic acids, stilbenes, and lignans. Flavonoids constitute the largest group of polyphenols and can be further classified into six subclasses: flavanols, flavonols, flavanones, flavones, anthocyanins, and isoflavones. Phenolic acids are mainly divided into: hydroxybenzoic and hydroxycinnamic acid. Polyphenols can exert a plethora of biological activities, including antioxidant, anti-inflammatory, anti-bacterial, and anti-proliferation activity, as well as enzyme and hormone regulation capacity. However, health effects of polyphenols depend on the quantity consumed and their bioavailability [11].

Current evidence from clinical trials suggests that polyphenol intake can reduce MetS by decreasing body weight, blood pressure, and blood glucose and by improving lipid metabolism. Chiva-Blanch et al. demonstrated a beneficial impact of red wine polyphenols (mainly catechin, epicatechin, malvidin-3-glucoside, gallic acid) on insulin resistance and lipid profile after 4 weeks’ intake in men at high cardiovascular risk [12]. Chen et al. showed that resveratrol capsules improves insulin resistance, glucose, and lipid metabolism after 3 months’ duration in patients with non-alcoholic fatty liver disease [13]. In a study by Martinez-Maqueda et al., dried grape pomace, which contained anthocyanins and favanols, lowered insulin resistance in MetS patients after a 6 week period [14]. However, a study by Tynkkynen et al. showed that epicatechin from chocolate increases HDL-cholesterol in nonsmoking volunteers [15]. Other studies underlined the significant role of anthocyanins from orange juice in lowering body mass index, waist circumference, and systolic and diastolic blood pressure in obese patients [16], as well as the role of gallic acid from cherry juice in lowering blood pressure and total- and LDL-cholesterol in older adults after 12 weeks’ intervention [17]. However, some clinical studies have shown inconsistent or controversial results. In the study by Janssens et al., a long-term tea extract (mainly catechin) supplementation did not exert a significant influence on body weight, body mass index, or fat deposition [18]. Additionally, in the study by Fang et al., a mango supplementation (full of gallotannin) did not decrease blood pressure in obese patients [19]. However, recent meta-analyses and systematic reviews of prospective cohort studies have confirmed that dietary polyphenols, especially flavonoids, play a role in the prevention of type 2 diabetes and cardiovascular diseases [20,21]. Therefore, taking into account the low bioavailability and metabolism, it should be emphasized that the protective functions of polyphenols can be effective only with long-term, regular consumption.

There is no special diet for all patients with MetS. The general assumptions for lifestyle changing should be based on WHO recommendations [22]. However, nutritional therapy and physical activity should be planned individually, depending on the patient’s health condition, dietary preferences, and country-specific foods rich in polyphenols.

Therefore, the aim of the study was to estimate how individual nutritional therapy affects the reduction of MetS risk factors in the early stage of the disease. We hypothesized that individual dietary counselling is an effective method of MetS treatment.

## 2. Materials and Methods

### 2.1. Ethical Approval

The study was conducted in accordance with the Helsinki Declaration and Good Clinical Practice and was approved by the Ethics Committee of the Medical University of Bialystok, Poland (No. R-I-002/227/2017 and R-I-002/382/2018). Informed consent was given by all participants of the study.

### 2.2. Subject Characteristics and Study Design

Subjects (35–65 years) diagnosed with MetS were recruited in the Lomza Medical Centre from February to June 2018. MetS was diagnosed when at least 3 of 5 risk factors were identified: (1) elevated waist circumference (≥94 cm for men and ≥80 cm for women); (2) elevated triglycerides (≥150 mg/dL); (3) reduced HDL-cholesterol (<40 mg/dL for men and <50 for women); (4) elevated blood pressure (systolic ≥ 130 and/or diastolic ≥ 85 mm Hg); (5) elevated fasting glucose (≥100 mg/dL, 5.6 mmol/L) [1].

**Figure 1:**
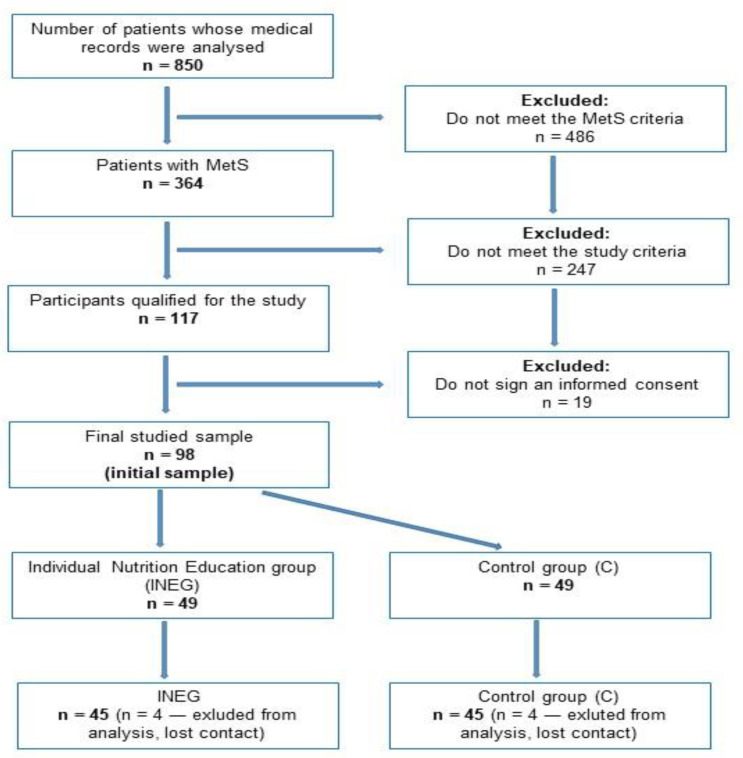
Flow-chart of study participants.

A flow-chart of study participants is shown in Figure 1. The initial study group consisted of 364 patients with MetS. After taking into account the exclusion criteria (age < 35 and > 65 years; taking anti-hypertensive, anti-hypercholesterolemia, and anti-hyperglycemia medications; long-term diabetes, hypertension, and dyslipidemia with complications; cancer; arteriosclerosis; cardiovascular incidents; pregnancy; lactation), 117 participants were qualified for the study. A total of 19 subjects did not sign an informed consent to participate in this study. The final analysis was conducted among 98 patients. The research was conducted within 3 months from July to September 2018. The study consisted of one intervention group and one control group. The individual nutrition education group (INEG) received full nutrition education and a nutrition plan, while the control group (CG) received standard care (general dietary recommendations provided by the dietician). Participants were randomly assigned to the INEG or CG. Randomization was stratified on the basis of age and gender. Each group was assigned 49 participants. After randomization, baseline data were collected from all the participants. The follow up was done after the 3 months intervention period. Finally, 4 patients from the INEG and 4 patients from the CG were excluded from analysis (lost contact).

### 2.3. Sociodemographic and Physical Activity Information

Sociodemographic characteristics (e.g., age, gender, education level, working status, marital status, smoking status) were collected from self-reported questionnaires. The physical activity was assessed by a short version of the International Physical Activity Questionnaire (IPAQ) [23]. On the basis of 7 questionnaire questions, the respondents were classified into the following categories: low, moderate, and high physical activity.

### 2.4. Dietary Assessment

Dietary assessments were performed by the dietician during the first visit and after 3 months’ intervention using 3-day 24-h dietary recalls (two randomly selected weekdays and one weekend day). Food portion sizes were estimated using an album with photographs of the most consumed Polish food products and dishes [24]. Energy and nutrient intakes from dietary assessments were calculated using the Diet 5.0 computer program, which uses a food composition database with over 3000 food products, dishes, and supplements commonly consumed in Poland. Nutrition knowledge was evaluated using the KomPAN questionnaire, created by the Behavioral Conditions of Nutrition Team, the Committee of Human Nutrition Science, and the Polish Academy of Science [25].

Dietary polyphenol intake (DPI) was determined by multiplying the daily consumption of individual food items by polyphenol contents in these food items. Data on the polyphenol content in foods were obtained from Polish polyphenol databases described in previous study [26], as well as the online Phenol-Explorer database [27].

### 2.5. Clinical Measurements

Anthropometric measurements, including body mass, height, waist circumference, and body composition, were performed by a dietician, using standardized procedures. Body mass index (BMI) was calculated as body mass in kilograms divided by squared height in meters (kg/m^2^) and interpreted according to WHO recommendations [28]. Body composition (fat mass, muscle mass) was measured by the analyzer InBody 230 (InBody Bldg, Seoul, Korea). Blood pressure was measured three times on the right arm in the sitting position after 15 min of rest at the one-minute intervals, using automatic devices Omron M3 (Genexo, Warsaw, Poland).

Blood biochemical tests (fasting glucose, LDL-cholesterol, HDL-cholesterol, triglycerides) were performed in an accredited laboratory of the Lomza Medical Centre, using enzymatic-colorimetric methods on the analyzer Cobas 6000 (Roche Diagnostics, Warsaw, Poland).

### 2.6. Intervention

Nutrition education intervention was conducted by a dietician in the INEG group. The individual intervention consisted of three nutrition education counselling sessions. Written dietary guidelines and lists of recommended and contraindicated products in the diet, with face-to-face lessons on MetS risk factors, were individually developed for each patient, depending on the occurrence of the following conditions: central obesity, elevated fasting glucose, elevated triglycerides, elevated blood pressure, and reduced high-density lipoprotein. At the end of the session, information and education materials were distributed. Additionally, the patients could contact a dietician throughout the study via phone or email. Follow up was done every 1 month during visits to the Medical Centre. Moreover, every week, the patient was followed up via a phone call to check the application of dietary recommendations and to provide additional information. The diet plan (10-day menu) was prepared by a dietitian, based on dietary guidelines for diabetes, obesity, and hypertensive and dyslipidemia patients [22,29,30], as well as guidelines in the line with the pyramid of healthy eating and physical activity for the Polish population (developed by the National Institute of Food and Nutrition) [31,32]. Additionally, polyphenol-rich foods were proposed [33,34] (Table 1). For overweight and obese patients, a reduction diet was recommended, taking into account 0.5 kg of weight loss per week. The 10-day menu was modified every month. All education sessions for participants were taken by the same dietitian. An example of an individual detailed dietary plan was presented in Supplementary Materials. The physical activity recommendation was given to the INEG (minimum 150 min of moderate intensity exercise each week, e.g., walking, cycling, aerobics).

### 2.7. Statistical Analysis

Statistical analyses were performed using Statistica 13.3 software (StatSoft Inc., Krakow, Poland). Continuous variables were presented as mean and standard deviation (SD) and categorical variables as count (N) and percentage (%). Categorical variables were compared with the Pearson’s chi-squared test. Normality of continuous data distribution was verified with the Shapiro–Wilk test and the Kolmogorov–Smirnov test with Lilliefors correction. Comparisons of continuous variables between groups were conducted using the Mann–Whitney–Wilcoxon or Kruscal–Wallis tests. Correlations were calculated by the Spearman rank test. *p*-values of less than 0.05 were considered statistically significant. Linear regression was used to investigate the relationship between dietary polyphenols and MetS risk factors post-intervention in the INEG. The one univariate and two multivariate models were specifically adjusted for age, gender, educational level, nutritional knowledge, physical activity, BMI, energy of diet, dietary fiber, and dietary fat.

## 3. Results

The baseline characteristics of the study participants (INEG and CG) are shown in Table 2. No significant differences were detected between groups in age, gender, education level, working status, marital status, physical activity, smoking, or nutritional knowledge. The mean age of the subjects was 45.5 ± 7.5 years in the INEG and 46.8 ± 10.7 years in the CG. In both groups, there was a similar number of men and women (55% of women and 45% of men in the INEG vs. 53% of women and 47% of men in the CG). Over 70% of the study participants were working and married, almost 90% had middle or high education level, and about 20% were smoking. Moreover, over 50% of subjects had unsatisfactory nutritional knowledge and over 60% were characterized by low physical activity.

**Figure 2:**
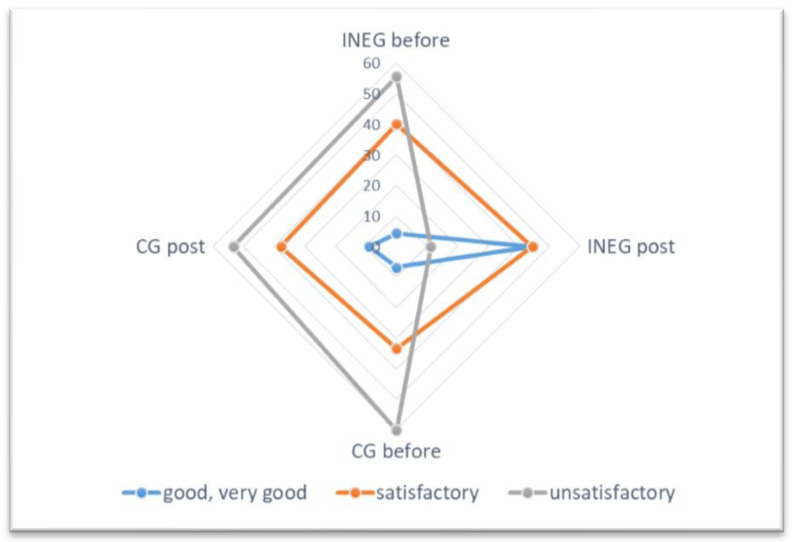
Assessment of nutrition knowledge among patients using the KomPAN questionnaire.

**Figure 3:**
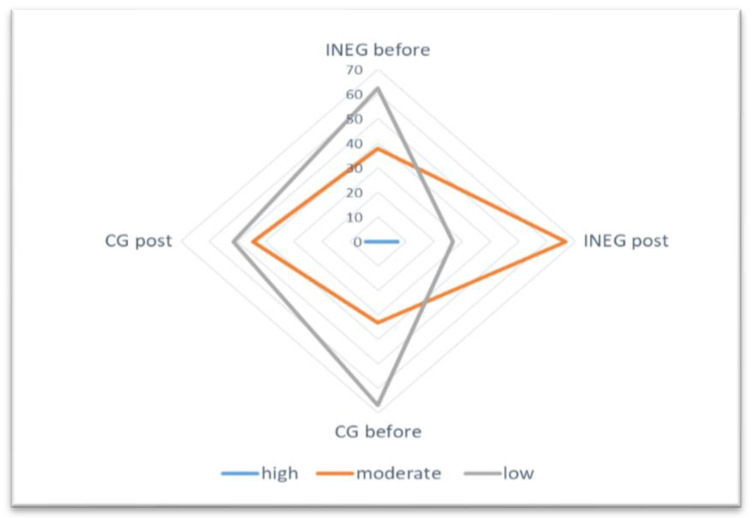
Assessment of physical activity among patients using the IPAQ questionnaire.

The results of the evaluation of nutrition knowledge and physical activity in the study groups are shown in Figure 2; Figure 3. It was found that nutritional knowledge and physical activity of the INEG was significantly (*p* < 0.001) higher post-intervention. No differences in nutrition knowledge or physical activity were shown for the CG before or post-intervention.

Changes in nutritional value of the diet before and post-intervention are presented in Table 3. There were no significant differences in the nutritional values of the diets between the INEG and CG before dietary intervention. The post-intervention INEG consumed lower amounts of SFA (*p* = 0.001) and higher MUFA (*p* = 0.001) and PUFA (*p* = 0.035), and the ratio of PUFA n-6:n-3 was lower (*p* = 0.001). In addition, post-intervention, the diet of INEG contained significantly less cholesterol (*p* = 0.009) and sodium (*p* = 0.001) and more fiber (*p* = 0.008), magnesium (*p* = 0.009), vitamin C (*p* = 0.008), total polyphenols (*p* = 0.001), total flavonoids (*p* = 0.011), flavanols (*p* = 0.009), anthocyanins (*p* = 0.001), and phenolic acids (*p* = 0.045). No differences were found in the CG diet before or post-intervention.

Changes in metabolic outcomes before and post-intervention are shown in Table 4. Before educational intervention, no significant differences in terms of metabolic outcomes between the INEG and CG were shown. Post-intervention, there was a significant decrease in body weight (*p* = 0.025), BMI (*p* = 0.009), waist circumference (*p* = 0.015), body fat mass (*p* = 0.012), fasting glucose (*p* = 0.009), total cholesterol (*p* = 0.012), and LDL-cholesterol (*p* = 0.008) and an increase in HDL-cholesterol (*p* = 0.018) in the INEG. No differences were found in the CG before or post-intervention.

Table 5 presents the prevalence of MetS risk factors before and post-intervention. It shows that post-intervention significantly (*p* = 0.001) decreased a number of participants with ≥3 MetS risk factors in the INEG. Significant improvement has been shown for following MetS risk factors: waist circumference (*p* = 0.011), fasting glucose (*p* = 0.009), and HDL-cholesterol (*p* = 0.007) in the INEG. No differences were found in the CG diet before or post-intervention.

Table 6 present correlations between dietary nutrients and improvement MetS risk factors post-intervention in the INEG. It was shown that WC and FG significantly (*p* < 0.05) correlated with fiber (r = −0.45, r = −0.48), polyphenols (r = −0.45, r = −0.54), flavonoids (r = −0.44, r = −0.65), flavanols (r = −0.43, r = −0.38), and anthocyanins (r = −0.41, r = −0.51), respectively. Whereas, HDL-C significantly (*p* < 0.05) correlated with SFA (r = −0.35), PUFA (r = 0.31), PUFA n-6:n-3 ratio (r = −0.41), fiber (r = 0.38), polyphenols (r = 0.43), flavonoids (r = 0.48), flavanols (r = 0.42), and anthocyanins (r = 0.45).

Table 7 shows the results of a linear regression analysis of dietary polyphenols and MetS risk factors. After the adjustments (model 3), we found significant association between dietary total polyphenols, flavonoids and anthocyanins, and fasting glucose, which explained 35%, 45%, and 32% of the variation, respectively.

## 4. Discussions

Metabolic syndrome significantly increases the risk of developing type 2 diabetes and cardiovascular disease. Proper nutrition is the most important action in the prevention and treatment of metabolic disorders, even before drug treatment. Nutrition education is a fundamental element to training patients in making healthy food choices.

The current study determined the effect of individual nutrition education on MetS in Polish patients. A diet plan, based on dietary guidelines for diabetes and cardiovascular disease prevention, as well as the pyramid of healthy eating and physical activity for the Polish population, was prepared individually for each patient. Dietary recommendations took into account patients’ dietary preferences and country-specific foods rich in polyphenols [22,29,30,31,32,33,34]. Moreover, individual physical activity was recommended, depending on the health condition.

It was found that the individual nutrition education intervention was an effective method to improve the knowledge, dietary habits, and physical activity of the study participants and had a significant effect on reducing some MetS risk factors.

Some studies have found that nutritional education improved nutritional knowledge but had no significant effect on the dietary practices of the young, healthy participants [35]. However, other studies have shown a beneficial effect of nutritional education in improving knowledge and eating habits in pregnant women [36]. In our study, after the intervention, an improvement was noted in nutritional knowledge and dietary practices of MetS participants. Similar to our findings, a large body of scientific evidence supports the effectiveness of individualized nutritional therapy in the treatment of metabolic disorders [37].

In this study, nutrition education did not affect overall fat intake but had an effect on the fatty acid profile. The post-intervention INEG consumed significantly less SFA and cholesterol and more PUFA and MUFA. Moreover, the ratio of n-6:n-3 in the INEG diet was 3:1 in comparison to the ratio 12:1 in the CG. At present, more emphasis is placed on the proportion of individual fatty acids in the diet than reducing overall fat consumption. PUFA n-3 from plant foods (linseed, walnuts) and fish have favorable effects on serum lipids. The dietary intake of SFA and cholesterol is an important determinant of plasma LDL-C levels, although some studies indicate that the food matrix (such as meat) plays a larger role in cardiovascular pathologies than the overall intake of SFA and cholesterol [38,39,40].

In the current study, lower intake of sodium and higher intake of magnesium, fiber, and antioxidants (polyphenols, vitamin C) were observed post-intervention. Previous studies have highlighted the important role of these dietary compounds in metabolic disorders [41,42].

Nutrition education programs conducted in other countries have shown the effectiveness of this method in reducing some MetS risk factors [43,44]. In our study, lower waist circumference, fat mass, fasting glucose, total cholesterol, and LDL-cholesterol and higher HDL-cholesterol showed post-intervention in the INEG. No differences were found in the CG before or post-intervention.

It was found that consumption of fiber and total polyphenols inversely correlated with abdominal obesity and fasting glucose.

The beneficial effect of dietary polyphenol intake on the reduction of some MetS risk factors has been shown in previous studies [45,46,47]. In the recent PREDIMED study, total polyphenol intake was associated only with a higher HDL-C but higher intake of stilbenes and lignans showed an inverse association with blood pressure, fasting glucose, and triglycerides. In this population study, polyphenol intake was relatively low (846 ± 318 mg/day) in comparison to our study (in the INEG post-intervention: 2089.6 ± 764.5) [45]. In the HAPIEE study, total dietary polyphenols and some classes of polyphenols (flavonoids: flavanols, flavanones, flavones, and anthocyanins, as well as stilbenes and phenolic acids) were associated with a lower risk of T2DM [46]. In our study, a linear regression analysis showed that, after adjustment for potential confounders, a significant impact on reducing fasting glucose had higher intake of total polyphenols, flavonoids, and anthocyanins. Polyphenols can reduce the T2DM risk by a number of mechanisms, including slowing carbohydrate digestion and glucose absorption, stimulation of insulin secretion, activation of insulin receptors and glucose uptake, influence on gene expression, and regulation of various tissue signaling pathways. In addition, polyphenols may reduce abdominal obesity by modulating physiological and molecular pathways involved in energy metabolism [47].

A previously published study showed that dietary fiber improved insulin resistance and glucose tolerance, reduced abdominal obesity, lowered total cholesterol, and decreased diastolic blood pressure. The mechanisms of action of dietary fiber on MetS risk factors are related to the reduction of the rate of nutrient absorption, suppression of appetite, regulation of energy homeostasis, and changes in the gut microbiome [48].

In our study, HDL-C was positively associated with fiber, polyphenols, PUFA, and PUFA n-3 and inversely with SFA. HDL-C is a lipoprotein with anti-atherogenic properties by reversing cholesterol transport from the peripheral tissues to liver. Low HDL-C, as well as high LDL-C, is associated with the development of CVD. A previous study indicated that consumption of fish containing n-3 PUFAs may be positively associated with HDL metabolism [49].

The present study also has some strengths and limitations. The strength of this study is that we provided individual nutritional counselling, depending on the patient’s health condition and the pyramid of healthy eating and physical activity for the Polish population. In addition, the diet plan (10-day menu) was corrected every month and took into account the patients’ food preferences and country-specific foods rich in polyphenols. The physical activity and nutrition knowledge were assessed by standardized questionnaires. Moreover, we used twice repeated 3-day 24-h dietary recalls to collect the data on nutrition from the Diet 5.0 computer program, developed by the National Institute of Food and Nutrition, to calculate the nutrition value of the diet. Finally, we had a low drop-out (8%) despite a relatively large group (n = 90) in comparison to other studies [50,51,52]. The main limitation of this study is that the study period was limited to 3 months, which only allowed assessment of short-term effects of the intervention. A longer study period is recommended to demonstrate long-term effects. However, the study period of 2–3 months is typical for this type of study [36,43].

## 5. Conclusions

The individual nutrition education was an effective method to improve the knowledge, dietary habits, and physical activity of the study participants. The modification of the diet in terms of higher intake of polyphenols (flavonoids and anthocyanins), fiber, and polyunsaturated fatty acids (PUFA) and lower intake of saturated fatty acids (SFA) had a significant impact on the improvement of some MetS risk factors (waist circumference, fasting glucose, and HDL-cholesterol). Therefore, individual nutrition education should be recommended for MetS patients before starting pharmacological treatment.

## Figures and Tables

**Table 1:** Characteristics of the recommended diet [22,29,30,31,32,33,34].

Dietary Components	Recommended Intake
Energy ^1^	1500–2500 kcal
Protein	20–25% of total energy
Fat	25–35% of total energy
Carbohydrates	45–50% of total energy
SFA	<7% of total energy
MUFA	15–20% of total energy
PUFA	6–10% of total energy
Cholesterol	<200 mg/day
Sodium	1500 mg/day
Fiber	≥25 g/day
Fruits and vegetables (without potatoes)	≥5 servings (500 g/day; 1/4 fruits, 3/4 vegetables)
Fish	≥2 servings/week (1 serving—150 g prepared for consumption)
Legumes	≥2 servings/week (1 serving—40–60 g dry)
Unsalted nuts and seeds	≥3–4 servings/week (1 serving—30–50 g)
Low-glycemic-index foods	GI < 55
Wholegrain cereal products	5 servings/day (1 serving—30 g bread or 30 g groats or pasta before cooking)
Meat	3 servings/week (1 serving—100–120 g prepared for consumption, preference for poultry > red meats)
Low-fat dairy products	3 servings/day (1 serving—200 mL of milk, 150 g yogurt or kefir, 35 g cottage cheese)
Oil (olive oils, linseed oil, rapeseed oil)	≥4 servings/ day (1 serving—1 tablespoon)
Eggs	4–5/week
Water	1.5–2 L/day
Polyphenol-rich foods	walnuts, sunflower seeds, raspberries, cranberries, blueberries, strawberries, beetroot, red cabbage
Sweet, cakes	not recommended
Average caloric distribution of meals	breakfast—25–30%, morning snack—5–10%, lunch—35–40%, afternoon snack—5–10%,dinner—15–20%
Frequency of eating meals	every 3–4 h

SFA—saturated fatty acids, MUFA—monounsaturated fatty acids, PUFA—polyunsaturated fatty acids. GI—glycemic index. ^1^ Energy of the diet was determined individually based on age, gender, body weight, physical activity, and the occurrence of metabolic disorders.

**Table 2:** Baseline characteristics of the participants.

Variable	INEG	CG	*p*-Value
Age (years), mean ± SD	45.5 ± 7.5	46.8 ± 10.7	0.415
Gender, N (%)			
women	25 (55.0)	24 (53.0)	0.865
men	20 (45.0)	21 (47.0)	
Education level, N (%)			
high	16 (35.6)	18 (40.0)	0.564
middle	23 (51.1)	22 (48.9)	
under middle	6 (13.3)	5 (11.1)	
Working status, N (%)			
currently working	35 (77.8)	33 (73.3)	0.486
Marital status, N (%)			
married	38 (84.4)	34 (75.6)	0.263
single	7 (15.6)	11 (24.4)	
Physical activity, N (%)			
high	0 (0.0)	0 (0.0)	0.543
moderate	17 (37.8)	15 (33.3)	
low	28 (62.2)	30 (66.7)	
Smoking status, N (%)			
currently smoking	10 (22.2)	9 (20.0)	0.496
Nutritional knowledge, N (%)			
Good, very good	2 (4.4)	3 (6.7)	0.658
satisfactory	18 (40.0)	15 (33.3)	
unsatisfactory	25 (55.6)	27 (60.0)	

Date are presented as mean ± standard deviation (SD) or number and percentage (N, %), INEG—individual nutrition education group, CG—control group.

**Table 3:** Changes in nutritional value of the diet at baseline and 3-months post-intervention.

Parameter	Before Intervention			Post Intervention			*P ^3^*	*P ^4^*
	INEG	CG	*P ^1^*	INEG	CG	*P ^2^*		
Energy (kcal)	2612.2 ± 345.8	2767.5 ± 376.3	0.564	2341.4 ± 437.3	2565 ± 374.9	0.256	0.324	0.385
Protein (% energy)	22.8 ± 7.4	20.6 ± 6.2	0.365	18.8 ± 8.7	20.3 ± 7.3	0.244	0.312	0.454
Fat (% energy)	34.5 ± 8.6	32.8 ± 9.5	0.465	31.2 ± 7.4	33.5 ± 6.9	0.157	0.487	0.515
SFA (% energy)	21.1 ± 12.5	19.2 ± 14.2	0.287	7.5 ± 5.4	16.7 ± 8.9	0.001	0.001	0.211
MUFA (% energy)	7.5 ± 4.6	8.1 ± 5.9	0.322	15.6 ± 7.1	8.5 ± 6.8	0.009	0.001	0.458
PUFA (% energy)	8.6 ± 5.9	7.7 ± 5.3	0.255	10.5 ± 5.9	8.1 ± 4.3	0.025	0.035	0.288
PUFA n-6:n-3	15:1	13:1	0.432	3:1	12:1	0.001	0.001	0.545
Cholesterol (mg)	444.1 ± 164.5	428.7 ± 148.7	0.195	256.4 ± 95.8	412.9 ± 126.4	0.001	0.009	0.234
Fiber (g)	15.6 ± 9.1	12.8 ± 10.4	0.255	27.4 ± 9.4	14.8 ± 10.7	0.001	0.008	0.195
Sodium (mg)	3150.4 ± 483.1	2923.5 ± 594.7	0.452	1785.2 ± 246.4	3150.4 ± 483.1	0.001	0.001	0.654
Magnesium (mg)	265.1 ± 115.4	286 ± 145.7	0.544	405.1 ± 215.4	304.2 ± 162.4	0.012	0.009	0.216
Calcium (mg)	822.4 ± 255.3	857.5 ± 247.2	0.456	951.3 ± 295.6	874.2 ± 264.4	0.065	0.086	0.451
Vitamin C (mg)	47.2 ± 25.4	41.9 ± 36.5	0.623	96.3 ± 36.5	52.4 ± 26.8	0.011	0.008	0.112
Vitamin A (µg)	825.8 ± 395.6	860.5 ± 412.3	0.235	850.6 ± 435.9	920.2 ± 571.6	0.134	0.312	0.298
Vitamin E (mg)	8.4 ± 3.2	7.2 ± 4.5	0.185	10.8 ± 4.8	8.5 ± 5.3	0.095	0.213	0.317
Vitamin B6 (mg)	1.5 ± 0.8	1.7 ± 0.5	0.115	1.8 ± 0.9	1.6 ± 0.6	0.115	0.244	0.367
Vitamin B12 (µg)	1.8 ± 1.1	2.0 ± 1.5	0.145	2.4 ± 1.8	2.1 ± 1.6	0.075	0.185	0.412
Folate (µg)	287.4 ± 144.5	311.5 ± 133.6	0.365	335.7 ± 214.5	295.2 ± 186.4	0.088	0.117	0.311
Polyphenols (mg)	1531.2 ± 481.5	1618.8 ± 511.6	0.186	2089.6 ± 764.5	1654.3 ± 543.2	0.001	0.001	0.468
Flavonoids	801.5 ± 387.3	780.2 ± 432.9	0.468	1125.8 ± 236.4	802.3 ± 452.1	0.008	0.011	0.345
flavanols	551.3 ± 295.9	531.6 ± 323.4	0.385	725.7 ± 195.8	553.6 ± 330.8	0.015	0.009	0.435
flavonols	91.4 ± 78.9	85.4 ± 85.3	0.411	108.7 ± 59.5	92.7 ± 75.8	0.114	0.098	0.105
flavanones	83.3 ± 72.7	79.8 ± 69.9	0.454	96.9 ± 55.2	82.9 ± 65.4	0.232	0.118	0.411
flavones	16.8 ± 11.8	15.7 ± 12.2	0.623	18.4 ± 8.5	16.6 ± 10.5	0.375	0.285	0.387
anthocyanins	31.8 ± 56.7	29.6 ± 42.4	0.585	105.7 ± 35.8	26.8 ± 54.2	0.001	0.001	0.395
isoflavones	2.1 ± 0.5	1.8 ± 0.7	0.314	2.9 ± 0.6	2.2 ± 0.9	0.354	0.323	0.423
Phenolic acids	695.5 ± 322.6	798.4 ± 345.2	0.285	896,7 ± 216.4	810.7 ± 314.8	0.087	0.045	0.564
hydroxybenzoic	85.2 ± 78.4	89.5 ± 80.8	0.456	95.7 ± 63.9	91.4 ± 82.1	0.245	0.115	0.476
hydroxycinnamic	602.4 ± 356.3	684.9 ± 423.7	0.325	784.6 ± 276.5	704.9 ± 388.5	0.312	0.095	0.392
Stilbenes	0.2 ± 0.8	0.2 ± 1.1	0.786	0.3 ± 0.5	0.2 ± 0.9	0.236	0.163	0.485
Lignans	0.5 ± 9.5	0.5 ± 10.2	0.644	0.6 ± 7.4	0.5 ± 9.7	0.354	0.322	0.678

Data are presented as mean ± standard deviation (SD) or number; INEG—individual nutrition education group, CG—control group, SFA—saturated fatty acids, MUFA—monounsaturated fatty acids, PUFA—polyunsaturated fatty acids. *P ^1^*—Statistical significance determined between the INEG and CG before intervention. *P ^2^*—Statistical significance determined between the INEG and CG post-intervention. *P ^3^*—Statistical significance determined between the INEG before and post-intervention. *P ^4^*—Statistical significance determined between the CG before and post-intervention.

**Table 4:** Changes in metabolic outcomes at baseline and 3-months post-intervention.

Parameter	Before Intervention			Post Intervention			*P ^3^*	*P ^4^*
	INEG	CG	*P* ^1^	INEG	CG	*P* ^2^		
Weight (kg)	97.6 ± 34.3	91.2 ± 29.2	0.155	82.5 ± 21.3	93.4 ± 31.7	0.011	0.025	0.356
BMI (kg/m^2^)	32.6 ± 12.2	30.2 ± 11.5	0.245	27.5 ± 5.4	29.4 ± 9.5	0.021	0.009	0.411
WC (cm)	105.3 ± 18.6	108.5 ± 15.8	0.432	91.1 ± 14.4	108.3 ± 18.6	0.009	0.015	0.455
MM (kg)	35.2 ± 7.2	34.1 ± 8.8	0.156	33.9 ± 9.5	32.7 ± 7.9	0.234	0.242	0.328
FM (kg)	36.4 ± 15.2	37.8 ± 13.5	0.387	28.4 ± 12.5	35.7 ± 18.7	0.034	0.012	0.265
FG (mg/dL)	107.6 ± 16.8	110.4 ± 19.4	0.432	89.5 ± 13.1	105.2 ± 17.2	0.011	0.009	0.324
TC (mg/dL)	232.3 ± 35.3	251.4 ± 29.8	0.115	185.5 ± 22.4	244.1 ± 27.6	0.009	0.012	0.565
LDL-C (mg/dL)	143.4 ± 25.3	148.4 ± 21.5	0.355	115.9 ± 16.5	142.8 ± 18.7	0.001	0.008	0.487
HDL-C (mg/dL)	54.6 ± 9.1	50.23 ± 11.3	0.231	65.8 ± 12.4	52.2 ± 9.5	0.025	0.018	0.314
TG (mg/dL)	169.8 ± 29.1	166.4 ± 25.2	0.554	148.2 ± 18.9	159.6 ± 21.5	0.085	0.068	0.249
SBP (mm Hg)	141.6 ± 13.4	138.8 ± 10.6	0.387	135.2 ± 11.8	145.1 ± 9.5	0.115	0.217	0.363
DBP (mm Hg)	89.4 ± 7.9	87.2 ± 10.5	0.425	84.6 ± 8.4	86.5 ± 9.8	0.225	0.378	0.512

Data are presented as mean ± standard deviation (SD); INEG—individual nutrition education group, CG—control group, BMI –body mass index, WC—waist circumference, MM—muscle mass, FM—fat mass, FG—fasting glucose, TC—total cholesterol, LDL-C—low-density lipoprotein, HDL-C—high-density lipoprotein, TG—triglycerides, SBP—systolic blood pressure, DBP—diastolic blood pressure. *P ^1^*—Statistical significance determined between the INEG and CG before intervention. *P ^2^*—Statistical significance determined between the INEG and CG post-intervention. *P ^3^*—Statistical significance determined between the INEG before and post-intervention. *P ^4^*—Statistical significance determined between the CG before and post-intervention.

**Table 5:** Prevalence of MetS risk factors at baseline and 3-months post intervention.

Parameter	Before Intervention			Post Intervention			*P ^3^*	*P ^4^*
	INEG	CG	*P ^1^*	INEG	CG	*P ^2^*		
Elevated WC	35 (77.8)	38 (84.4)	0.556	27 (60.0)	39 (86.7)	0.008	0.011	0.432
Elevated FG	30 (66.7)	27 (60.0)	0.452	17 (37.8)	28 (62.2)	0.001	0.009	0.395
Elevated TG	15 (33.3)	18 (40.0)	0.397	13 (28.9)	15 (33.3)	0.422	0.565	0.483
Reduced HDL-C	13 (28.9)	15 (33.3)	0.622	8 (17.8)	14 (31.1)	0.012	0.007	0.388
Elevated BP	26 (57.8)	28 (62.2)	0.458	27 (60.0)	29 (64.4)	0.452	0.354	0.411
MetS risk factors								
1–2	0 (0.0)	0 (0.0)	-	28 (62.2)	2 (4.4)	0.001	0.001	0.254
3	30 (66.7)	34 (75.6)	0.233	15 (33.3)	31 (68.9)	0.001	0.001	0.315
4–5	15 (33.3)	11 (24.4)	0.185	2 (4.4)	12 (26.7)	0.001	0.001	0.455

Data are presented as number and percentage (N, %), elevated WC—≥94 cm for men and ≥80 cm for women, elevated FG—≥100 mg/dl, elevated TG—≥150 mg/dl, reduced HDL-C—<40 mg/dl for men and <50 for women, elevated BP—systolic ≥130 and/or diastolic ≥85 mm Hg; INEG—individual nutrition education group, CG—control group, WC—waist circumference, FG—fasting glucose, HDL-C—high-density lipoprotein, TG—triglycerides, BP—blood pressure. *P ^1^*—Statistical significance determined between the INEG and CG before intervention. *P ^2^*—Statistical significance determined between the INEG and CG post-intervention. *P ^3^*—Statistical significance determined between the INEG before and post-intervention. *P ^4^*—Statistical significance determined between the CG before and post-intervention.

**Table 6:** Correlations between dietary nutrients and MetS risk factors post-intervention in the INEG.

Nutrients in Diet	WC	FG	HDL-C
SFA (% energy)	0.08	0.07	−0.35 *
MUFA (% energy)	−0.16	−0.12	−0.14
PUFA (% energy)	−0.09	−0.11	0.31 *
PUFA n-6:n-3	−0.11	−0.19	−0.41 *
Cholesterol (mg)	0.13	0.09	−0.15
Fiber (g)	−0.45 *	−0.48 *	0.38 *
Sodium (mg)	0.02	0.17	0.01
Magnesium (mg)	−0.01	−0.06	0.11
Vitamin C (mg)	−0.08	−0.12	0.14
Polyphenols (mg)	−0.45 *	−0.54 *	0.43 *
Flavonoids (mg)	−0.44 *	−0.65 *	0.48 *
Flavanols (mg)	−0.43 *	−0.38 *	0.42 *
Anthocyanins (mg)	−0.41 *	−0.51 *	0.45 *
Phenolic acids (mg)	−0.21	−0.19	0.22

* *p* < 0.05; INEG—individual nutrition education group, CG—control group, WC—waist circumference, FG—fasting glucose, HDL-C—high-density lipoprotein, SFA—saturated fatty acids, MUFA—monounsaturated fatty acids, PUFA—polyunsaturated fatty acids.

**Table 7:** Univariate and multivariate linear regression models between dietary polyphenols and MetS risk factors post-intervention in the INEG.

Model	Variables	WC	FG	HDL-C
	**Polyphenols (mg)**			
1	β (95% CI) *p*-value	−0.392 (0.085–0.624) 0.014	−0.535 (0.287–0.756) 0.006	−0.388 (0.097–0.624) 0.012
	Model parameters	*R^2^* = 0.11	*R^2^* = 0.44	*R^2^* = 0.10
2	β (95% CI) *p*-value	−0.355 (0.085–0.582) 0.021	−0.498 (0.185–0.722) 0.011	−0.374 (0.105–0.549) 0.011
	Model parameters	*R^2^* = 0.10 0.025	*R^2^* = 0.38 0.014	*R^2^* = 0.09 0.044
3	β (95% CI) *p*-value	−0.402 (0.095–0.712) 0.014	−0.482 (0.195–0.698) 0.019	−0.397 (0.118–0.564) 0.013
	Model parameters	*R^2^* = 0.06 0.225	*R^2^* = 0.35 0.024	*R^2^* = 0.09 0.112
	**Flavonoids (mg)**			
1	β (95% CI) *p*-value	−0.422 (0.142–0.654) 0.011	−0.652 (0.398–0.912) 0.001	−0.476 (0.155–0.698) 0.012
	Model parameters	*R^2^* = 0.15	*R^2^* = 0.57	*R^2^* = 0.14
2	β (95% CI) *p*-value	−0.414 (0.112–0.622) 0.014	−0.632 (0.355–0.897) 0.001	−0.442 (0.201–0.623) 0.016
	Model parameters	*R^2^* = 0.13 0.021	*R^2^* = 0.55 0.001	*R^2^* = 0.11 0.034
3	β (95% CI) *p*-value	−0.387 (0.106–0.698) 0.034	−0.597 (0.311–0.815) 0.001	−0.398 (0.125–0.612) 0.045
	Model parameters	*R^2^* = 0.09 0.095	*R^2^* = 0.45 0.009	*R^2^* = 0.08 0.114
	**Flavanols (mg)**			
1	β (95% CI) *p*-value	−0.422 (0.112–0.654) 0.017	−0.373 (0.085–0.545) 0.016	−0.392 (0.154–0.523) 0.024
	Model parameters	*R^2^* = 0.12	*R^2^* = 0.14	*R^2^* = 0.11
2	β (95% CI) *p*-value	−0.388 (0.089–0.565) 0.021	−0.352 (0.115–0.512) 0.025	−0.376 (0.117–0.526) 0.019
	Model parameters	*R^2^* = 0.10	*R^2^* = 0.11	*R^2^* = 0.12
3	β (95% CI) *p*-value	−0.361 (0.079–0.584) 0.022	−0.335 (0.086–0.554) 0.031	−0.344 (0.115–0.545) 0.039
	Model parameters	*R^2^* = 0.09 0.134	*R^2^* = 0.06 0.219	*R^2^* = 0.08 0.185
	**Anthocyanins (mg)**			
1	β (95% CI) *p*-value	−0.394 (0.135–0.711) 0.011	−0.498 (0.195–0.798) 0.001	−0.435 (0.155–0.681) 0.015
	Model parameters	*R^2^* = 0.13	*R^2^* = 0.39	*R^2^* = 0.14
2	β (95% CI) *p*-value	−0.351 (0.118–0.668) 0.014	−0.477 (0.211–0.744) 0.001	−0.415 (0.132–0.654) 0.018
	Model parameters	*R^2^* = 0.11 0.019	*R^2^* = 0.35 0.011	*R^2^* = 0.16 0.034
3	β (95% CI) *p*-value	−0.335 (0.113–0.598) 0.024	−0.455 (0.178–0.699) –0.015	−0.382 (0.098–0.591) 0.021
	Model parameters	*R^2^* = 0.08 0.119	*R^2^* = 0.32 0.015	*R^2^* = 0.09 0.135

Model 1: univariate analysis; Model 2: multivariate analysis adjusted for age and gender; Model 3: multivariate analysis adjusted for age, gender, educational level, nutritional knowledge, physical activity, BMI, energy of diet, dietary fiber, and dietary fat; CI—coefficient interval.

## Supplementary Materials

The following are available online at https://www.mdpi.com/article/10.3390/nu13062102/s1, An example of an individual detailed dietary plan.
