# Supplementary material for: Individual Diet Modification Reduces the Metabolic Syndrome in Patients Before Pharmacological Treatment
Source: Nutrients. 2021 Jun 19;13(6):2102. doi: 10.3390/nu13062102 (PMC8234117; doi:10.3390/nu13062102)
Supplement: Supplementary file 1 [file nutrients-13-02102-s001.zip › nutrients-1234892-supplementary.pdf]

Appendix S1

An example of a diet prepared for an overweight women with elevated waist circumference, elevated fasting glucose and elevated blood pressure.
The diet includes patient-preferred food products available in popular stores.

Gender: Woman
Age: 40 years old
Height: 160 cm
Weight: 70 kg
Body mass index (BMI): 27,3 kg/m^2
Waist circumference: 88 cm
Fasting glucose: 110 mg/dL
Blood pressure: 135/ 88 mm Hg
A reduction diet with a low glycemic index and a low glycemic load with a caloric content of about 1500 kcal was applied. The patient should consume 1.5 – 2.0 L of water daily.

DAY 1

BREAKFAST

AVOCADO AND TOMATO SALAD

avocado – 70 g<br>tomatoes, red – 200 g<br>olive oil – 8 g<br>garlic – 6 g<br>fresh lemon juice – 5 g<br>coriander, dried leaves – 1 g<br>coriander seeds – 1 g<br>black pepper – 1 g<br>graham bread – 60 g<br>cucumber – 110 g<br><br>chamomile infusion – 250 ml<br>water – 300 ml	1. Wash the avocado and tomatoes, cut into small cubes and mix.<br>2. Add pepper, crushed coriander seeds and coriander leaves, lemon juice and chopped garlic.<br>3. Lastly, pour in the olive oil.<br>4. Serve the finished salad on bread together with cucumber.

LUNCH

VEGETABLES WITH YOGURT DIP

red pepper, sweet – 75 g<br>cucumber – 100 g<br>natural yogurt, 1.5% fat – 150 g + flaxseed<br>herbs – 2 g<br><br>water – 300 ml	1. Chop the vegetables.<br>2. Eat with yogurt mixed with flaxseed and herbs.

DINNER

PASTA SALAD WITH TURKEY AND VEGETABLES

wholegrain wheat pasta – 30 g<br>broccoli – 105 g<br>red pepper, sweet – 75 g<br>garlic – 10 g<br>onion – 35 g<br>chives - 8 g – dressing<br>cold pressed rapeseed oil – 10 g<br>fresh lemon juice – 5 g<br>black pepper – 1 g<br>feta cheese – 10 g<br>walnuts – 10 g<br>turkey breast meat, skinless – 100 g<br>apple – 100 g<br><br>water – 300 ml	1. Bake the turkey in foil in the oven (approx. 20-30 min, 180 °C).<br>2. After it has cooled down, cut it into cubes.<br>3. Cook whole meal pasta, preferably penne until it is al dente.<br>4. Boil the broccoli.<br>5. Cut the peppers, onions, and garlic, then gently fry over 1/2 teaspoon of oil. Add broccoli, pasta, feta, and nuts.<br>6. Serve the whole thing with dressing.<br>7. After a meal, eat an apple.

AFTERNOON SNACK

VANILLA CHEESE WITH APRICOTS

curd cheese skinny – 75 g<br>natural yogurt, 1.5% fat – 40 g<br>apricots – 80 g<br>non-alcoholic vanilla extract – 3 g<br>water – 300 ml	1. Mix curd cheese with yogurt and vanilla extract.<br>2. Add the chopped fruit.

SUPPER

PANCAKES SERVED WITH HOMEMADE HUMMUS

unroasted buckwheat – 30 g	1. Rinse unroasted buckwheat under running water.

water – 90 g<br>universal rapeseed oil – 8 g	2. Pour into a bowl and pour 2 cups of fresh water.<br>3. Leave for the night.<br>4. In the morning, pour out the unabsorbed water, add fresh water, and mix until a smooth mass. Dose the water carefully so that the dough is not too rare. You may not have to use the whole glass, unless you decide otherwise.<br>5. Add oil into the mass and mix thoroughly.<br>6. Fry the pancakes in a well-heated frying pan.
**Traditional hummus paste**	
canned chickpeas – 40 g<br>sesame butter, tahini – 2 g<br>fresh lemon juice – 4 g<br>garlic – 1.8 g<br>olive oil – 4 g<br>paprika – 2 g<br>water – 10 g<br><br>water – 300 ml	1. Mix chickpeas, tahini paste, lemon juice, and garlic until the mass is uniform. If it is too dense, add water.<br>2. Add spices and, lastly, pour olive oil.
**SUMMARY OF THE DAY**	
fat – 60.9 g ǀ total carbohydrates – 170.0 g ǀ fiber – 28.7 g ǀ cholesterol – 74.6 mg ǀ sodium – 743.2 mg ǀ protein – 77.6 g ǀ SFA – 9.5 g ǀ MUFA – 31.8 g ǀ PUFA – 15.8 g ǀ	

DAY 2

BREAKFAST

**YOGURT WITH FRUIT**	
natural yogurt, 2% fat – 150 g<br>banana – 40 g<br>walnuts – 10 g<br>blueberries – 80 g<br>apple – 150 g<br>curd cheese – 35 g<br><br>water – 300 ml	1. Mix yogurt with curd cheese and nuts.<br>2. Add the fruit.

LUNCH

**SANDWICHES WITH PASTE FROM DRIED TOMATOES AND VEGETABLES**	
parsley, leaves – 3 g<br>garlic – 6 g<br>sundried tomatoes, in oil, drained – 20 g<br>graham bread – 30 g<br>cucumber – 60 g<br>olive oil – 4 g<br><br>chamomile infusion – 250 ml<br>water – 300 ml	1. In a food processor, grind dried tomatoes, garlic and pour olive oil from dried tomatoes.<br>2. The mass should be ground until a smooth consistency is obtained.<br>3. The finished paste should be spread onto bread and served with vegetables.

DINNER

**COD IN TOMATOES SERVED WITH GROATS**	
cod, fresh, fillets without skin – 150 g<br>paprika – 4 g<br>tomatoes, red, ripe, cooked – 100 g<br>garlic – 6 g<br>dried oregano – 3 g<br>dried basil – 3 g<br>lemon juice – 15 g	1. The fish should be drizzled with lemon juice, seasoned with herbs and laid in a heatproof dish drizzled lightly with oil.<br>2. To prepare the sauce, pour a tablespoon of oil into the pot, fry the peeled and half-chopped garlic cloves, add the canned tomatoes, season with paprika, a teaspoon of dried oregano and a pinch of basil. Mix,

universal rapeseed oil – 10 g<br>bare groats, pearled – 60 g<br>tomatoes, red – 285 g<br>parsley, leaves – 4 g<br>olive oil – 8 g<br>mozzarella, low in sodium – 10 g<br><br>water – 300 ml	boil. Cook without covering for about 10 minutes until the tomatoes evaporate.<br>3. Preheat the oven to 200 °C.<br>4. Pour the reduced tomato sauce over the fish and place slices of tomato, parsley and, mozzarella cheese on top.<br>5. Place in preheated oven. Bake for 15-20 minutes.
**AFTERNOON SNACK**	
**SALAD WITH CROUTONS**	
graham bread – 30 g<br>mix of lettuce, smart (Fit&easy) – 50 g<br>red pepper, sweet – 60 g<br>olive oil – 5 g<br><br>water – 300 ml	1. Make bread croutons.<br>2. Chop the vegetables.<br>3. Mix the oil with any type of herbs.
**SUPPER**	
**MACKEREL PASTE SANDWICHES**	
mackerel, smoked – 50 g<br>onion – 15 g<br>chives – 5 g<br>chili powder – 2 g<br>graham bread – 30 g<br>olive oil – 8 g<br>curd cheese, skinny – 35 g<br>lemon balm infusion – 250 ml<br><br>water – 300 ml	1. Tear the fish into small chunks, mixed with onions, chives, olive oil, and cottage cheese. Season to taste with herbs and chili.<br>2. Serve on bread.
**SUMMARY OF THE DAY**	
fat – 63.3 g ǀ total carbohydrates – 181.9 g ǀ fiber – 29.1 g ǀ cholesterol – 128.8 mg ǀ sodium – 1389.0 mg ǀ protein – 82.7 g ǀ SFA – 11.9 g ǀ MUFA – 31.2 g ǀ PUFA – 15.3 g ǀ	
**DAY 3**	
**BREAKFAST**	
**QUINOA WITH APPLE MOUSSE**	
quinoa – 40 g<br>drinking milk, 2% fat – 200 g<br>apple – 100 g<br>rapeseed oil – 6 g<br><br>black coffee – 250 ml<br>water – 300 ml	1. Rinse the quinoa and cook in milk according to the given instructions on the package, with the addition of a teaspoon of oil.<br>2. Roast an apple on a teaspoon of water, add cinnamon to taste.<br>3. Serve the ready-made quinoa with apple mousse.
**LUNCH**	
**STRAWBERRY SALAD**	
spinach – 100 g<br>strawberries – 100 g	1. Wash the spinach and strawberries, tear the spinach and break up berries as you like.<br>2. Combine with dressing.
**Raspberry dressing**	
raspberries – 50 g<br>maple syrup – 10 g<br>balsamic vinegar – 10 g<br>water – 20 g<br>black pepper – 3 g	1. Raspberries should be chopped and boiled in water in a saucepan for about 10 minutes.<br>2. Next, add maple syrup, oil, balsamic vinegar, ginger, and black pepper.

rapeseed oil – 4 g<br>ginger root – 2 g<br><br>water – 300 ml	3. The sauce is ready when it becomes thick – about 10 min.
**DINNER**	
**GRILLED CHICKEN SALAD**	
arugula – 10 g<br>salad mix, party (Fit&easy) – 40 g<br>olive oil – 8 g<br>walnuts – 15 g<br>chicken breast meat, without skin – 100 g<br>smoked paprika – 3 g<br>lemon juice – 20 g<br>whole grain rye bread – 60 g<br>avocado – 50 g<br><br>chamomile infusion – 250 ml<br>water – 300 ml	1. Chop vegetables as you wish.<br>2. Make bread croutons.<br>3. Clean the meat from the chicken breast, gently break down and roll in spices: smoked paprika, any type of herbs. Grill in a dry pan.<br>4. Combine ready-made salad ingredients with dressing and avocado.
**AFTERNOON SNACK**	
**SALAD**	
cherry tomatoes – 100 g<br>arugula – 10 g<br>red pepper, sweet – 75 g<br>salad mix, party (Fit&easy) – 50 g<br>cold pressed rapeseed oil – 6 g<br>chicken breast meat, skinless – 30 g<br><br>water – 300 ml	1. Chop the vegetables.<br>2. Grill the chicken breast in herbs, then cut into cubes.<br>3. Combine the salad ingredients with rapeseed oil.
**SUPPER**	
**COTTAGE CHEESE SANDWICHES**	
wholegrain rye bread – 60 g<br>curd cheese – 70 g<br>arugula – 10 g<br>olive oil – 8 g<br>chives – 8 g<br><br>water – 300 ml	1. Mix cottage cheese with olive oil and chives.<br>2. In addition, you can season to taste with herbs.<br>3. Serve whole on bread.
**SUMMARY OF THE DAY**	
fat – 63.4 g | total carbohydrates – 168.0 g | fiber – 33.2 g | cholesterol – 95.3 mg | sodium – 835.1 mg | protein – 76.5 g | SFA – 9.8 g | MUFA – 31.6 g | PUFA – 17.7 g |	

DAY 4

**BREAKFAST**	
**PORRIDGE WITH APPLE AND FLAXSEED**	
oatmeal – 40 g<br>drinking milk, 2% fat – 200 g<br>apple – 100 g<br>flaxseed – 15 g<br><br>black coffee – 250 ml<br>water – 300 ml	1. Roast the oatmeal in a dry saucepan, then add milk, flaxseed, and apple.<br>2. Boil everything until the flakes gently soften.
**LUNCH**	
**YOGURT WITH RASPBERRIES AND ALMONDS**	

natural yogurt, 2% fat – 150 g<br>raspberries – 50 g<br>almonds, cereals – 8 g<br><br>water – 300 ml	Mix raspberries and almonds with yogurt.

DINNER

VEGETABLE CURRY

chickpeas without deluge, rinsed under tap water – 80 g<br>unroasted buckwheat – 60 g<br>zucchini – 110 g<br>white mushroom – 100 g<br>garlic – 10 g<br>onions – 70 g<br>olive oil – 8 g<br>lemon zest, fresh – 2 g<br>chives – 3 g<br>ground turmeric – 3 g<br>paprika – 3 g<br>pumpkin seeds – 10 g<br>smoked paprika – 2 g<br>hot chili pepper, sun dried – 2 g<br><br>water – 300 ml	1. Chop vegetables as you wish.<br>2. Pour a small amount of oil into a deep frying pan and fry chopped mushrooms and zucchini, add garlic squeezed through the press.<br>3. Pour out the water and rinse chickpeas under running water on a strainer and then add it to the pan.<br>4. Simmer for 10-15 minutes.<br>5. After that, add lemon zest and spices.<br>6. The finished dish is served with unroasted buckwheat, sprinkled with pumpkin seeds and chives.

AFTERNOON SNACK

TOASTS WITH COTTAGE CHEESE PASTE

curd cheese skinny – 40 g<br>onion – 20 g<br>dill – 6 g<br>black pepper – 2 g<br>graham bread – 50 g<br>red pepper, sweet – 75 g<br><br>water – 300 ml	1. Curd cheese should be mixed with dill, onions, pepper, and chopped pepper.<br>2. Serve the finished paste on bread.

SUPPER

TURKEY AND AVOCADO SIRLOIN SALAD

avocado – 70 g<br>red pepper, sweet – 75 g<br>tomatoes, red – 110 g<br>olive oil – 8 g<br>salad mix, smart (Fit&easy) – 100 g<br>turkey tenderloin – 40 g<br><br>chamomile infusion – 250 ml<br>water – 300 ml	1. Chop salad ingredients as you wish.<br>2. Combine everything with olive oil seasoned with herbs.

SUMMARY OF THE DAY

fat – 62.7 g | total carbohydrates – 205.9 g | fiber – 39.9 g | cholesterol – 49.6 mg | sodium – 965.4 mg | protein – 71.4 g | SFA – 12.2 g | MUFA – 30.5 g | PUFA – 15.2 g |

DAY 5

BREAKFAST

SANDWICHES WITH VEGETABLE PASTE

tomatoes, red – 100 g<br>eggplant – 100 g	1. Clean the vegetables, then drizzle with oil, season with pepper, basil, garlic, smoked paprika, and chili.

red pepper, sweet – 75 g<br>onion – 40 g<br>sundried tomatoes, in oil, drained – 12 g<br>olive oil – 8 g<br>fresh basil – 5 g<br>garlic – 3 g<br>black pepper – 1 g<br>chili cayenne pepper – 1 g<br>smoked paprika – 2 g<br>oregano dried – 1 g<br>graham bread – 60 g<br>sunflower seeds – 5 g<br><br>black coffee – 250 ml<br>water – 300 ml	2. Bake prepared vegetables in an oven preheated to 200 ^{o}C for about 30 minutes.<br>3. After baking and a slight cooling, the vegetables should be peeled and finely cut.<br>4. Serve the finished paste with bread sprinkled with sunflower seeds.
**LUNCH**	
**YOGURT WITH STRAWBERRIES**	
natural yogurt for drinking, 2% fat – 150 g<br>strawberries – 100 g<br>ground cinnamon – 2 g<br>graham bread – 30 g<br><br>water – 300 ml	1. Add fruits and cinnamon to yogurt.<br>2. Eat with graham bread.
**DINNER**	
**PASTA WITH VEGETABLE SAUCE AND TURKEY**	
buckwheat noodles – 40 g<br>turkey breast meat, without skin – 100 g<br>spinach – 100 g<br>sundried tomatoes, in oil, drained – 40 g<br>onions – 70 g<br>garlic – 3 g<br>black pepper – 3 g<br>rosemary – 1 g<br>smoked paprika – 1 g<br>chili – 1 g<br>sunflower seeds – 15g<br>tomatoes, red, canned, stewed – 265g<br><br>green tea, infusion – 250 ml	1. On a sip of water, sauté the onion and garlic. When gently glazed, add spinach and simmer to reduce water.<br>2. In a dry Teflon frying pan, grill the turkey, diced and seasoned with herbs, pepper, and paprikas. When the turkey is browned, add dried tomatoes and canned tomatoes. Cook until the sauce is reduced, then add the previously evaporated spinach.<br>3. Ready-made sauce to serve with pasta can be sprinkled with sunflower seeds.
**AFTERNOON SNACK**	
**COTTAGE CHEESE WITH VEGETABLES**	
universal rapeseed oil – 8 g<br>red pepper, sweet – 75 g<br>curd cheese – 70 g<br>dill – 3 g<br>parsley, leaves – 4 g<br>chives – 8 g<br>sweet smoked paprika – 1 g<br><br>water – 300 ml	1. Cut the peppers julienne style,.<br>2. Mix cottage cheese with oil, dill, chives and parsley.<br>3. Season the finished cottage cheese with sweet smoked paprika and serve with red pepper.
**SUPPER**	
**PEAR SALAD**	
salad mix, party (Fit&easy) – 100 g<br>tomatoes, red – 110 g<br>avocado – 40 g	1. Chop the vegetables.<br>2. Make graham bread croutons.<br>3. Mix olive oil, parsley leaves, dill, lemon juice.

graham bread – 30 g<br>olive oil – 8 g<br>parsley, leaves – 4 g<br>dill – 8 g<br>lemon juice – 10 g<br>sunflower seeds – 5 g<br>pear – 65 g<br><br>water – 600 ml	4. Wash the pear, cut into thin slices and arrange on pre-prepared vegetables. Pour the whole dressing and mix together with croutons and sunflower seeds.

SUMMARY OF THE DAY

fat – 61.1 g ǀ total carbohydrates – 206.1 g ǀ fiber – 40.1 g ǀ cholesterol – 62.4 mg ǀ sodium – 1522.2 mg ǀ protein – 78.2 g ǀ SFA – 9.3 g ǀ MUFA – 31.7 g ǀ PUFA – 15.4 g ǀ

DAY 6

BREAKFAST

AVOCADO PASTE SANDWICHES

avocado – 60 g<br>lemon juice – 15 g<br>olive oil – 4 g<br>garlic – 10 g<br>black pepper – 1 g<br>graham bread – 60 g<br>sunflower seeds – 15 g<br>cucumber – 100 g<br><br>green tea, infusion – 250 ml<br>water – 300 ml	1. Peel the avocado and crush with a fork.<br>2. Add lemon juice, pepper, and crushed garlic.<br>3. Mix thoroughly and, lastly, add olive oil.<br>4. Serve the ready-made paste on bread with cucumber and sunflower.

LUNCH

SWEET CURD CHEESE SANDWICHES

curd cheese – 70 g<br>graham bread – 30 g<br>maple syrup – 10 g<br>blueberries – 50 g<br>water – 300 ml	1. Mix cottage cheese with maple syrup.<br>2. Serve on bread with blueberries.

DINNER

FISH WITH VEGETABLES SERVED WITH GROATS

salt, fresh – 150 g<br>onions – 35 g<br>black pepper – 1 g<br>olive oil – 8 g<br>yellow green beans, boiled, soaked, with salt – 150 g<br>universal rapeseed oil – 15 g<br>dill – 3 g<br>parsley – 3 g<br>fresh basil – 3 g<br>oatmeal – groats – 30 g<br><br>black, coffee – 250ml<br>water – 300 ml	1. Clean the fish, season with pepper, dill pour oil and arrange the onion slices.<br>2. Bake in 180°C under cover for 15-20 minutes.<br>3. In the meantime, cook the beans al dente.<br>4. Serve the finished fish with groats and green beans mixed with olive oil and seasoned with herbs.

AFTERNOON SNACK

COCTAIL

apple – 100 g<br>pear – 65 g<br>natural yogurt, 2% fat – 150 g	All ingredients should be mixed until the mass is uniform.

water – 300 ml	

SUPPER

PASTA WITH BAKED VEGETABLES

wholegrain wheat pasta, cooked – 70 g<br>zucchini – 110 g<br>onions – 35 g<br>garlic – 12 g<br>tomatoes, red, canned, stewed – 125 g<br>black pepper – 1 g<br>parsley, leaves – 4 g<br>olive oil – 4 g<br>feta cheese – 10 g<br>pumpkin seeds – 10 g<br><br>water – 300 ml	1. On the baking sheet from the oven, put the chopped garlic.<br>2. Drizzle with oil, season, stir, and put in the oven in 240 ^{o}C for 30 min.<br>3. After the first 15 min of baking, stir and re-insert for another 15 min.<br>4. Cook the pasta al dente.<br>5. Put the vegetables in the pot, add tomatoes and spices. Cook the whole thing over low heat about 5-10 min.<br>6. Serve prepared sauce with pasta sprinkled with feta and pumpkin seeds.

SUMMARY OF THE DAY

fat – 65.6 g | total carbohydrates – 180.9 g | fiber – 30.5 g | cholesterol – 97.3 mg | sodium – 1455.1 mg | protein – 81.5 g | SFA – 10.8 g | MUFA – 34.3 g | PUFA – 15.8 g |

DAY 7

BREAKFAST

BAKED SALMON SANDWICHES,TOMATO EGG IN LEMON SAUCE

wholegrain rye bread – 55 g<br>olive oil – 10 g<br>salmon, baked – 60 g<br>cucumber – 100 g<br>chives – 8 g<br>lemon juice – 5 g<br>tomatoes, red – 110 g<br>chicken eggs, whole – 40g<br><br>water – 300 ml	1. Bake salmon in 180°C for 15-25 minutes in herbs: dill, garlic, parsley.<br>2. Mix the oil with lemon juice and chives. Add tomatoes and cucumbers to the dressing.<br>3. Serve the finished salmon with hard-boiled egg, bread, and a salad.

LUNCH

SWEET COTTAGE CHEESE

curd cheese – 70 g<br>blueberries – 100 g<br>flaxseed – 5 g<br><br>water – 300 ml	Mix all ingredients.

DINNER

SALMON AND VEGETABLES SERVED WITH GROATS

barley groats, boiled – 90 g (30 g dry)<br>parsley, leaves – 4 g<br>onion – 70 g<br>garlic – 3 g<br>olive oil – 5 g<br>dried thyme – 3 g<br>salmon, Atlantic, wild, fresh – 150 g<br>black pepper – 2 g<br>dried oregano – 2 g<br>zucchini – 285 g<br><br>green tea, infusion – 250ml	1. Cook the barley groats according to the instructions on the package.<br>2. In a heatproof dish, arrange the salmon, add the onion slices, zucchini, garlic squeezed through the press, olive oil, and herbs. Bake in 180°C for about 25-35 minutes.<br>3. Serve the ready-made salmon with barley.

water – 300 ml	
**AFTERNOON SNACK**	
**COCTAIL**	
kefir, 1.5% fat – 150 g<br>apple – 100 g<br>walnuts – 5 g<br><br>water – 300 ml	Mix all ingredients.
**SUPPER**	
**SANDWICHES WITH ARUGULA PESTO**	
wholegrain rye bread – 65 g<br>red pepper, sweet – 75 g<br>arugula – 10 g<br>olive oil – 10 g<br>sundried tomatoes, in oil, drained – 15 g<br><br>water – 300 ml	1. Blend arugula, garlic, and dried tomatoes until a uniform mass, adding olive oil.<br>2. Spread the pesto onto the bread and add red peppers
**SUMMARY OF THE DAY**	
fat – 61.9g ǀ total carbohydrates – 163.9 g ǀ fiber – 30.1 g ǀ cholesterol – 290.1 mg ǀ sodium – 836.7 mg ǀ protein – 90.4g ǀ SFA – 11. 9 g ǀ MUFA – 29.3 g ǀ PUFA – 16. 2 g ǀ	

DAY 8

**BREAKFAST**	
**AVOCADO AND COTTAGE CHEESE SANDWICHES**	
graham bread – 55 g<br>avocado – 50 g<br>curd cheese – 70 g<br>olive oil – 3 g<br>chives – 8 g<br>radish – 50 g<br>red pepper, sweet – 80 g<br><br>lemon balm infusion – 250 ml<br>water – 300 ml	Spread cottage cheese mixed with olive oil and chives on the bread, then add vegetables and avocado.
**LUNCH**	
**QUICK SALAD**	
red pepper, sweet – 75 g<br>lettuce – 50 g<br>cucumber – 110 g<br>olive oil – 10 g<br>lemon juice – 15 g<br>honey – 5 g<br><br>water – 300 ml	1. Chop the vegetables as you wish.<br>2. Combine with dressing: olive oil, lemon juice, and honey.
**DINNER**	
**ROAST TROUT SERVED WITH GROATS AND QUICK SALAD, VEGETABLE SOUP**	
carrots – 70 g<br>parsley, root – 25 g<br>leek – 30 g<br>celery – 30 g<br>water – 350 g<br>universal rapeseed oil – 6 g<br>onions – 40 g	1. Chop vegetable as you wish.<br>2. On a small amount of oil fry the onions and spices, then add the remaining vegetables.<br>3. When the vegetables are gently fried, add water and bring to a boil.<br>4. Vegetables should be boiled until soft. In the meantime, you can season the soup with herbs.

cauliflower, frozen – 40 g<br>green beans, frozen – 50 g<br>dill – 2 g<br>parsley, leaves – 1 g<br>black pepper – 3 g<br>dried marjoram – 3 g<br>bay leaf – 3 g<br>whole allspice – 3 g<br>jet trout, fresh – 150 g<br>chili powder – 2 g<br>smoked paprika – 2 g<br>parsley – 8g<br>garlic – 3 g<br>olive oil – 6 g<br>roasted buckwheat – 50 g<br>mix of lettuce with arugula (Vital Fresh) – 50 g<br>tomatoes, cherry – 100 g<br><br>green tea, infusion – 250 ml<br>water – 300 ml	5. Trout should be cleaned and seasoned with chilies, parsley, garlic, and smoked paprika. Bake in briquette in 180 °C for about 15-25 minutes.<br>6. Serve the finished trout with buckwheat and a quick tomato salad.

AFTERNOON SNACK

COCTAIL

kefir, 1.5% fat – 150 g<br>flaxseed – 2 g<br>apple – 100 g<br>blueberry – 100 g<br><br>water – 300 ml	Mix all ingredients until a smooth mass.

SUPPER

TOMATO PASTE SANDWICHES

sundried tomatoes, in oil, drained – 30 g<br>sunflower oil – 4 g<br>sunflower seeds, shelled seeds, dry roasted, without salt – 15 g<br>black pepper – 1 g<br>sweet paprika, ground – 1 g<br>graham bread – 50 g<br>red pepper, sweet – 75 g<br><br>water – 300 ml	1. The sunflower seeds should be roasted in a heated frying pan, then mixed until a smooth mass with tomatoes and sunflower oil.<br>2. Season the finished paste to taste with sweet paprika and smoked paprika.<br>3. Serve on bread with red pepper.

SUMMARY OF THE DAY

fat – 62.4 g | total carbohydrates – 198.5 g | fiber – 43.0 g | cholesterol – 95.9 mg | sodium – 926.4 mg | protein – 81.7 g | SFA – 9.3 g | MUFA – 30.4 g | PUFA – 17.9 g |

DAY 9

BREAKFAST

SCRAMBLED EGGS SERVED WITH CREAM CHEESE SANDWICHES WITH VEGETABLES

whole chicken eggs – 40 g<br>chicken egg white – 100 g<br>universal rapeseed oil – 10 g<br>whole grain rye bread – 60 g<br>creamy cheese (e.g. Philadephia), with reduced fat – 10 g<br>red pepper, sweet – 75 g	1. Mix egg whites and an egg until a uniform mass, season to taste with black pepper.<br>2. Fry in a frying pan with a little oil.<br>3. Serve scrambled eggs with sandwiches with cream cheese and vegetables.

cucumber – 110 g<br><br>water – 300 ml	

LUNCH

VEGETABLES WITH COTTAGE CHEESE, APPLE

curd cheese skinny – 55 g<br>red pepper, sweet – 100 g<br>arugula – 40 g<br>linseed oil – 5 g<br>salad mix, family (FIt&Easy) – 70 g<br>apple – 80 g<br>parsley leaves – 8 g<br>chives – 3 g<br>black pepper – 1 g<br><br>black coffee – 250 ml<br>water – 300 ml	1. Mix cottage cheese with linseed oil and spices.<br>2. Serve with vegetables.

DINNER

ROAST COD SERVED WITH GROATS AND SALAD

cod, fresh – 130 g<br>lemon – 40 g<br>dill – 8 g<br>garlic – 3 g<br>parsley, leaves – 4 g<br>roasted buckwheat – 50 g<br>cucumber – 120 g<br>olive oil – 12 g<br>pumpkin, seeds – 6 g<br>salad mix, cool (Fit&Easy) – 100 g<br>chives – 3 g<br>fresh basil – 10g<br><br>water – 300 ml	1. Clean the cod and place it in a briquette together with slices of lemon, dill, garlic, parsley.<br>2. Bake in 180 ^{o}C for about 30-35 minutes.<br>3. Serve the cod with buckwheat and salad: cucumber cut into slices, combined with a mix of lettuce and olive oil with herbs.<br>4. Sprinkle the whole dish with pumpkin seeds.

AFTERNOON SNACK

FUITS WITH SEEDS

pumpkin, seeds – 10 g<br>apple – 140 g<br>strawberries – 100 g<br>water – 300 ml	Cut apples into larger cubes and mix with the remaining ingredients.

SUPPER

COTTAGE CHEESE SALAD

avocado – 30 g<br>arugula – 15 g<br>sundried tomatoes, in oil, drained – 15 g<br>wholegrain rye bread – 40 g<br>olive oil – 6 g<br>salad mix, party (Fit&easy) – 100 g<br>curd cheese – 50 g<br><br>lemon balm infusion – 250 ml<br>water – 300 ml	1. Mix olive oil with dried tomatoes.<br>2. Cut the avocado into slices, arrange on a mix of lettuce and arugula.<br>3. Arrange the curd cheese on the vegetables and pour dressing with dried tomatoes on top.<br>4. Serve with bread.

SUMMARY OF THE DAY

fat – 60.8 g | total carbohydrates – 175.5 g | fiber – 35.1 g | cholesterol – 217.5 mg | sodium – 1053.1 mg | protein – 89.8 g | SFA – 9.6 g | MUFA – 29.6 g | PUFA – 16.2 g |

DAY 10

BREAKFAST

SANDWICHES WITH HERBAL PASTE	
curd cheese skinny – 70 g<br>natural yogurt, 2% fat – 30 g<br>chives – 8 g<br>white pepper – 3 g<br>wholegrain rye bread – 60 g<br>red pepper, sweet – 75 g<br>olive oil – 8 g<br><br>black coffee – 250ml<br>water – 300 ml	1. Cottage cheese should be mixed with chives, yogurt, and olive oil.<br>2. Season the paste, serve with bread and peppers.

LUNCH

SALAD WITH COTTAGE CHEESE	
tomatoes, red – 80 g<br>olive oil – 8 g<br>curd cheese, loose – 35 g<br>radish – 20 g<br>chives – 8 g<br>whole grain rye bread – 30 g<br>fresh basil – 10 g<br>oregano – 1 g<br><br>water – 300 ml	1. Chop the vegetables as you wish.<br>2. Mix the oil with basil, chives and season with pepper and oregano.<br>3. Pour the dressing over the vegetables and serve with bread.

DINNER

ZUCCHINI PIES SERVED WITH VEGETABLE SAUCE	
wheat flour, type 1850 – 30 g<br>zucchini – 100 g<br>whole chicken eggs – 40 g<br>onions – 35 g<br>dill – 8 g<br>universal rapeseed oil – 8 g<br>water – 150 ml	1. Pour the flour into a bowl, mix with freshly ground pepper, then with a beaten egg (preferably with a whisk). Pour water and mix it into a uniform dough.<br>2. Weight out the necessary amount of zucchini. Wash, dry, cut off the ends, and grate on large meshes. Do the same with onions. Gently squeeze zucchini and onions in your hands from excess water.<br>3. Add the zucchini and onions and chopped greens to the dough and mix with a spoon.<br>4. Heat the pan with oil. Apply a dose of mass each time and fry the pancakes for about 2-3 minutes. When browned, turn over to the other side and fry for about 2 more minutes.
**VEGETABLE SAUCE FOR PIES**	
canned chickpeas without deluge – 40 g<br>red peppers, sweet – 50 g<br>zucchini – 60 g<br>tomatoes, red, canned, stewed – 100 g<br>onion – 20 g<br>brown mushroom e.g. crimini – 40 g<br>olive oil – 8 g<br>smoked paprika – 1 g<br>ground paprika – 1 g<br>chili cayenne pepper – 1 g<br>black pepper – 1 g<br>dried marjoram – 1 g	1. Cut the onion into half-moons and fry in olive oil.<br>2. Add canned chickpeas, mushrooms, and fry together<br>3. Then, add the diced red peppers and all the spices. Simmer for 10 min.<br>4. After 10 minutes, add the chopped zucchini and tomatoes.<br>5. Season the dish.<br>6. Serve with zucchini pies and mozzarella cheese.

mozzarella, low in sodium – 20 g<br><br>water – 300 ml	

AFTERNOON SNACK

SIRLOIN SALAD	
red peppers, sweet – 75 g<br>olive oil – 6 g<br>parsley, leaves – 4 g<br>turkey tenderloin – 40 g<br>salad mix, party (Fit&Easy) – 30 g<br>maple syrup – 5 g<br><br>green tea, infusion – 250 ml<br>water – 300 ml	1. Crush the vegetables.<br>2. Combine olive oil with maple syrup and parsley.<br>3. Mix vegetables with dressing and sirloin.

SUPPER

YOGURT WITH FRUITS AND NUTS	
natural yogurt, 2% fat – 75 g<br>walnuts – 13 g<br>apple – 100 g<br>wholegrain rye bread – 60 g<br>watermelon – 100 g<br><br>water – 300 ml	1. Chop the fruit as you like and combine with the sauce.<br>2. Serve with bread.

SUMMARY OF THE DAY
fat – 65.2 g ǀ total carbohydrates – 189.5 g ǀ fiber – 33.1 g ǀ cholesterol – 187.6 mg ǀ sodium – 1518.0 mg ǀ protein – 72.4 g ǀ SFA – 12.5 g ǀ MUFA – 32.4 g ǀ PUFA – 15.5 g ǀ
